# Homogenous Chaotic Network Serving as a Rate/Population Code to Temporal Code Converter

**DOI:** 10.1155/2014/476580

**Published:** 2014-03-23

**Authors:** Mikhail V. Kiselev

**Affiliations:** Megaputer Intelligence Ltd., Office 403 Building 1, 69 Bakuninskaya Street, Moscow 105082, Russia

## Abstract

At present, it is obvious that different sections of nervous system utilize different methods for information coding. Primary afferent signals in most cases are represented in form of spike trains using a combination of rate coding and population coding while there are clear evidences that temporal coding is used in various regions of cortex. In the present paper, it is shown that conversion between these two coding schemes can be performed under certain conditions by a homogenous chaotic neural network. Interestingly, this effect can be achieved without network training and synaptic plasticity.

## 1. Introduction

Nervous system codes information in form of sequences of spikes or spike trains. Therefore, analysis of information processing in the brain is impossible without understanding principles of information coding and principles of conversion between different coding schemes, because it is well known that nervous system uses different coding schemes for transmitting information about stimuli, patterns, muscular commands, and so on. These coding schemes are based on two main approaches. In the first class of coding methods, the exact relative position of different spikes on the time axis is not taken into account, only their frequency or the sets of neurons emitting them are important. On the contrary, the other coding methods utilize exact delays between individual spikes. Let us call these two classes of codes asynchronous and synchronous codes.

There are several schemes of asynchronous coding and they are often used in combination. Rate coding is used in many afferent and efferent parts of nervous system. In this approach, intensity of a stimulus or command sent to a muscle is represented as number of spikes per unit time. It is the most explored coding method. Another asynchronous coding method, population coding, is based on representation of a stimulus as episodes of increased activity of a certain neuronal ensemble specific for this stimulus. It may be used to code the fact of presence of some stimulus as well as its strength, as a number of active neurons. For example, it is known [1] that visual image moving direction is encoded as activity of the respective neuronal ensembles in middle temporal visual area of primates' brain. Rate and population coding can be considered as two sides of the general coding scheme when presence and/or intensity of some stimulus is expressed by increasing firing rate in certain population of neurons. We will call this scheme rate/population coding. In a specific variant of this scheme (sometimes referred to as position coding), numeric parameter of a stimulus is coded as position of the most active neurons in the ensemble. It was noted that this type of coding has a number of advantages compared to rate coding [2].

Synchronous coding (usually called temporal coding [3]) is based on the idea that precise relative timing of individual spikes inside spike trains emitted by different neurons can contain information about stimuli. This representation may have different forms. The fact of presence of some stimulus can be represented as a stable combination of spikes emitted by certain neurons with fixed delays of spikes with respect to other spikes (and therefore we can call this kind of temporal coding the spatiotemporal coding). Continuous value can be encoded as a time interval between two spikes, as a phase shift between two spike trains, or a spike phase relative to some global synchronizing signal. For example, hippocampal CA1 pyramidal cells code body spatial location by their firing phase relatively to theta rhythm [4]. This coding method can be called the phase coding. The fact that temporal resolution of the neural code often has millisecond order of magnitude [5] is an evidence of wide usage of temporal coding in the brain. Most commonly used synaptic plasticity model, spike-timing dependent plasticity (STDP [6]), assumes this information coding scheme. Models of working memory based on neuronal polychronization effect [7, 8] are also naturally based on this coding method. Let us note that in this paper we will consider spatiotemporal variant of temporal coding only.

At present, it is evident that different sections of the nervous system utilize different information coding schemes. Primary afferent information encoded using rate- or population-based schemes is passed for processing to the cortex zones where temporal coding is widely used. But commands to muscles again should be represented as rate coded signals. It is also very probable that future intelligent systems and devices based on spiking neural networks (SNNs), for example, in robotics, will include components using various coding schemes. Therefore, SNNs performing functions of converter between different information coding forms should be a necessary part of nervous system as well as of these devices. However, in contrast with vast literature devoted to information coding in SNNs, the number of works considering conversion between different coding schemes is surprisingly few. For example, in [9] it was discussed how cortical bursting neurons could translate phase information contained in precisely timed spike sequences into rate coded signal. Relationship between rate and phase coding schemes in ensembles of hippocampal pyramidal neurons and translation from former to latter was explored in [10]. The question about which kind of networks could perform translation from rate/population coding to spatiotemporal coding seems to remain insufficiently explored until now. General approach to solution of this problem was presented by Izhikevich in [11] in relation to the so-called polychronous neuronal groups (PNGs). The idea is that desired conversion is performed by polychronous neuronal groups (populations of neurons which are being activated emit spike trains with precisely reproduced delays between individual spikes) existing or spontaneously emerging in chaotic neural network. However, this work as well as the subsequent works devoted to polychronization neither considered, to the extent of my knowledge, concrete conditions under which this conversion could be realized nor reported an experimental evidence of its realization. Achievement of these goals was motivation for the research reported here. The present work also uses polychronization effect as a basis, like [11], but, besides that, as we will see, it is shown in it that the network performing conversion from rate/population coding to spatiotemporal coding

1. may consist of leaky integrate-and-fire (LIF) neurons which are much simpler than the neuron model used in [11],
2. may not be plastic (while STDP plasticity was used in [11], it was noted there that synaptic plasticity can be harmful because it makes the conversion unstable),
3. does not need to be involved in global rhythmic activity like theta rhythm in [4].

## 2. Materials and Methods

In this research we utilized one of the simplest and in the same time the most widely used neuron model, leaky integrate-and-fire (LIF) neuron with absolute refractory period (see, e.g., [6]).

There are two kinds of neurons in the network: excitatory and inhibitory neurons. Axons of the excitatory and inhibitory neurons are connected only to excitatory or inhibitory synapses of other neurons, respectively. It is essential that postsynaptic spike emission is a result of collective activity of sufficient number (we set this number equal to 6) of presynaptic neurons. In order to use dimensionless units, we assume that the threshold membrane potential value is always equal to 1. To satisfy the above mentioned condition, the maximum excitatory synaptic weight value was selected equal to 0.19. Every individual synaptic weight was randomly selected using uniform distribution in the range (0, 0.19). Inhibitory synapse weights were also assigned with randomly generated values uniformly distributed in the range (0, *W* ^−^). Value of the maximum inhibitory weight *W* ^−^ was used as a regulator necessary for maintaining balance of excitation and inhibition in the network while numbers of inhibitory and excitatory neurons were fixed. Namely, we used the network consisting of 700 excitatory and 300 inhibitory neurons in all the experiments. In case of small *W* ^−^ even slight input signal or infrequent spontaneous firing causes avalanche of spike emissions leading the network to the state of constant self-sustaining activity with very high firing frequency. Great *W* ^−^ values cause immediate suppression of network reaction to any external signal. For every network configuration there exists a threshold value of *W* ^−^ above which self-sustaining network activity is impossible under any conditions. We selected value of *W* ^−^ slightly higher than this threshold. In our case, it corresponded to *W* ^−^ = 10.

Time of spike propagation from neuron to neuron lied in the range 1–10 ms for excitatory connections and 1–3 ms for inhibitory connections that is close to physiological values. Setting the spike propagation delays is considered below.

For sake of generality, the network should not have any structure a priori taking into account properties of input signal. In fact, the considered network has no intrinsic structure at all; it is completely homogenous and chaotic in the sense that all neurons of the same kind and all connections between the same kinds of neurons (excitatory → excitatory, excitatory → inhibitory, etc.) have the same distributions of weights, delays, connection probabilities, and so forth. Besides, neuron's axon can never be connected to a synapse of the same neuron.

Source of external signals received by the network is an array of its* input nodes*. Neurons are connected to them via excitatory or inhibitory synapses. Through these connections (we will call them afferent connections) neurons receive the signal consisting of noise (random spikes with constant mean frequency) and stimuli represented as short episodes of high frequency spiking of certain groups of input nodes. The total number of input nodes was always the same and was equal to 1000. Ratio of excitatory and inhibitory input nodes was the same as for neurons, 700/300.

Provided that the described conditions are met, selection of sets of presynaptic neurons and input nodes was absolutely random for every neuron.

Many SNN computer simulation experiments show that distribution of synaptic delays is an equally important factor determining network behavior as distribution of synaptic weights. For example, it is crucial that the propagation delay of inhibitory connections would be substantively less than of excitatory connections; it is necessary to prevent development of the powerful permanent global oscillatory network activity which can suppress network reaction to external stimuli (as it follows, e.g., from theoretical model considered in [12]). However, if this requirement is satisfied, the exact distribution of inhibitory connection delays does not influence network properties significantly. On the contrary, selection of excitatory connection delays was found to be very important so that we consider it more in detail. As it was mentioned above, in our approach the key role in realization of rate/population to temporal coding conversion is played by PNGs. In [8], it was proposed to use SNNs artificially enriched by potential PNGs due to specially tuned excitatory propagation delays. Namely, excitatory neurons were considered as located at random points on surface of sphere or N-dimensional hypersphere and connection delays were made proportional to the spherical distance between the neurons connected. Since PNGs are characterized by great number of short paths between the same pair of neurons such that the total delay in every path is (almost) identical, this distribution of delays gives much greater number of PNGs than totally random distribution. Similar to [8], in this work we used 4-dimensional sphere neuron placement. Experimental results considered in next section confirm importance of this choice.

The input signal consisted of sequence of different stimuli mixed with noise. Every stimulus was 30 ms long and was presented after network reaction to previous stimulus that faded away completely (that was achieved due to proper selection of *W* ^−^—as was discussed earlier). Every stimulus was a sequence of randomly generated spikes from set of input nodes corresponding to this stimulus. The stimuli were characterized by significantly higher spike frequency comparatively to the background noise.

Advantage of the LIF neuron model is that it is very simple. Model of its soma includes only two parameters: the length of refractory period *T* and the membrane potential decay constant *τ*. The former limits the maximum firing frequency and is usually selected equal to few milliseconds. We set *T* = 6 ms. The latter determines the size of time period during which arriving presynaptic spikes act together to produce postsynaptic spike. This parameter varies in different kinds of neurons [6], however, obviously, it cannot be great in neurons forming PNGs which are based on very exact firing timings. For this reason we set it to 3 ms.

The general structural properties of the network are summarized in Table 1.

**Figure 1:**
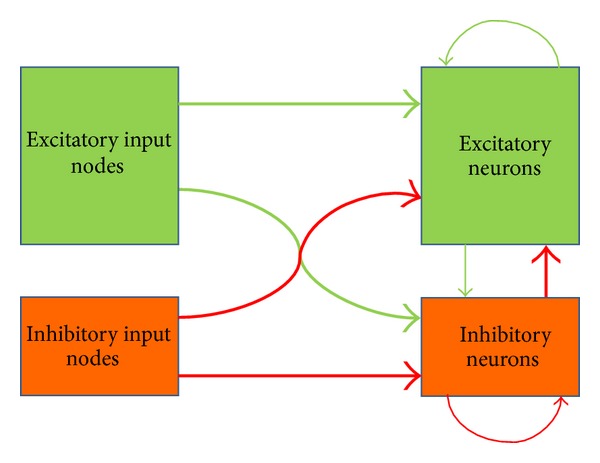
Excitatory (green) and inhibitory (red) neurons, input nodes, and synaptic connections. Size of blocks corresponds to relative amounts of neurons and input nodes. Thickness of arrows reflects total effective weights of the respective connections.

**Figure 2:**
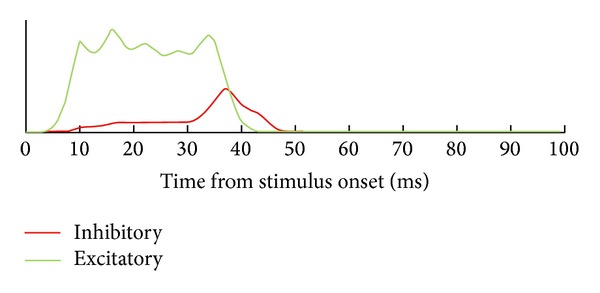
Dynamics of mean firing rate of excitatory (green) and inhibitory (red) neurons after stimulus presentation. Stimulus duration is 30 ms.

This table displays another important feature of the described network related to the role of inhibitory neurons. These neurons should not block network response to external stimuli but should efficiently stop network self-sustaining activity after end of stimulation. To reach this goal, the inhibitory neurons are themselves strongly inhibited by afferent signals, so that they almost do not fire during stimulation. But their mutual inhibition is much weaker than their inhibitory effect on the excitatory neurons (they have only 3 nonafferent inhibitory synapses, while excitatory neurons have 10). Therefore, just after the stimulus end the inhibitory neurons begin to fire extensively and suppress the whole network activity. The relative strength of excitation and inhibition in the network is shown schematically on Figure 1. The effect of this distribution of interneuron connections is depicted on Figure 2. It shows averaged firing frequency of excitatory and inhibitory neurons at different moments after beginning of stimulus presentation.

Now let us return to the final goal of this work. We see that the input signal is encoded in form of increased firing frequency of certain populations of the network input nodes. If every presentation of some stimulus activates a PNG specific for this stimulus it means, in our approach, that this stimulus is recoded to temporal form, since neurons belonging to active PNG fire in strict sequence with exact timings. Therefore, pursuing our goal we should solve the problem of finding PNGs in the network, and, in particular, the PNGs specific for the given patterns.

Basically, there are two different approaches to determination of PNGs inside a neural network [13]. The first method is based on analysis of the network structural properties such as synaptic delays and weights. In the second method the recordings of firing times of neurons are analyzed in order to determine stable repeating time-locked sequences of spikes associated with active PNGs. We used the second approach but implemented an alternative algorithm for PNG detection.

In my terms, PNG is defined by a sequence (neuron id, firing time). Only excitatory neurons are included in PNGs. Let us consider the stimulus *A*. Let *P*_*Ai*_ be a set of pairs (neuron id, time after the beginning of *i*th presentation of the stimulus *A*) corresponding to all spikes emitted before presentation of next stimulus. Set of all such sets corresponding to the stimulus *A* will be denoted as *𝒫*_*A*_ = {*P*_*Ai*_}. Then the algorithm for finding in *𝒫*_*A*_ a PNG with support *n*(*G*(*𝒫*_*A*_, *n*)) is the following:

1. create the matrix *C*_*at*_, initializing by 1*s* all its elements, for which 〈*a*, *t*〉 ∈ *P*_*A*1_, and by 0*s*, all the rest elements.
2. Iteratively for each *i*, 2 ≤ *i* ≤ *N*_*A*_, find the shift *s*, for which the value of ∑_〈*a*,*t*+*s*〉∈*P*_*Ai*__ *C*_*at*_ is the greatest, then increment by 1 those *C*_*at*_, for which 〈*a*, *t* + *s*〉 ∈ *P*_*Ai*_.
3. If ∀*t*∀ *aC*_*at*_ < *n*, then *G*(*𝒫*_*A*_, *n*) = *∅*, else if $\overset{˘}{t}$ is the least *t*, for which *C*_*at*_ ≥ *n*, then $\langle a,t-\overset{˘}{t}\rangle \in G({𝒫}_{A},n)\Leftrightarrow C_{at}\geq n$.

**Figure 3:**
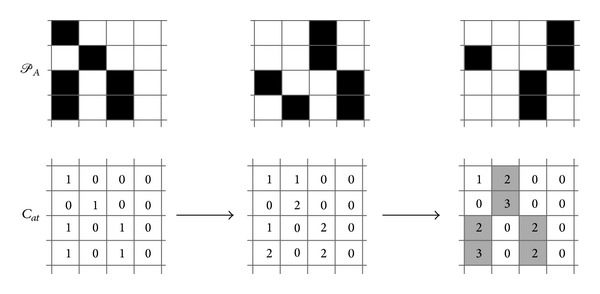
Determination of PNG with support 2 on 3 fragments of firing history of 4 neurons.

This algorithm can be illustrated by the following simple example (Figure 3). The upper row of this figure represents 3 fragments of firing history of 4 neurons (*x*-axis represents time), each fragment includes 4 time steps. Filled squares denote firing. The lower row displays the matrix *C*_*at*_ after step 1 of the algorithm and after 2 iterations on step 2. Gray squares in its final variant correspond to *G*(*𝒫*_*A*_, 2).

Using this algorithm the PNGs *G*(*𝒫*_*A*_, *n*) are found. But we are interested only in the PNGs specifically reacting to only one stimulus. Let us define activity of the PNG *G* in the history fragment *P*_*Ai*_ as *A*_*Ai*_(*G*) = max⁡_*s*_⁡|shift(*G*, *s*)∩*P*_*Ai*_|, where shift(*G*, *s*) is built from *G* by addition of *s* to second elements in all pairs in *G*. Then the strength of reaction of *G* to the stimulus *A* can be defined as *R*_*A*_(*G*) = min⁡_*i*_⁡*A*_*Ai*_(*G*), and the measure of selectivity of *G*(*𝒫*_*A*_, *n*) as *S*(*A*, *n*) = *N*_*A*_/|{〈*B*, *i*〉 : *A*_*Bi*_(*G*(*𝒫*_*A*_, *n*)) ≥ *R*_*A*_(*G*(*𝒫*_*A*_, *n*))}|. It is evident that if reaction of *G*(*𝒫*_*A*_, *n*) to any stimulus different from *A* is weaker than *R*_*A*_(*G*(*𝒫*_*A*_, *n*)), then *S*(*A*, *n*) = 1. It can be readily seen that the algorithm determining PNGs and their selectivity has complexity *O*(*TN*_*F*_ *N*_*S*_ *N*_*P*_^2^), where *T* is time between presentation of consecutive stimuli, *N*_*F*_ is number of all stimuli presentations, *N*_*S*_ is total number of spikes in the whole firing protocol analyzed, and *N*_*P*_ is number of different stimuli.

Recoding process was declared as successful if for every stimulus a PNG with selectivity 1 was found. For every PNG we recorded its size and strength of its reaction to the corresponding stimulus (in the relative units, divided by the size of this PNG). The latter parameter has meaning of the minimum part of the PNG becoming active after stimulus presentation. Besides that, it is important to know to what degree these PNGs are independent; how the fact that a neuron that belongs to one PNG changes probability to find it inside some other PNG. If PNGs are independent it means that network has enough informational capacity to be able to convert greater number of different stimuli without loss of accuracy. Also, in case of numerous stimuli, independent PNGs are less similar and therefore permit more reliable recognition of encoded stimulus. To characterize the degree of independence of two PNGs we use sets of neurons belonging to them, *S*_1_ and *S*_2_. If the PNGs are independent then size of the intersection of *S*_1_ and *S*_2_ equals approximately (|*S*_1_||*S*_2_|)/*N* ^+^, where *N* ^+^ is the total number of excitatory neurons. As a measure of dependence between two PNGs we take the ratio of real size of their intersection and this value. Proximity of the calculated number to 1 is an indication of their independence.

In all described experiments, determination of PNGs and measurement of their parameters were performed for 100 presentations of every stimulus. Results of these experiments are discussed in next section.

## 3. Results and Discussion

In the discussed experiments we varied 3 basic parameters of the input signal: number of different stimuli, intensity of stimuli (in terms of spike rate and population size), and signal/noise ratio. Only stimulus duration was always equal to 30 ms. It is close to duration of shortest stimuli recognized by living neural systems [14, 15].

The main goal of these experiments is to demonstrate that the desired conversion can be performed by the network described in previous section and that the effect is stable and observed under wide range of conditions, not just for carefully prepared specific signal. For this purpose it is quite sufficient to use star experiment design scheme starting from some point and varying different parameters separately; here we are not interested in exact dependencies of conversion characteristics on signal parameters, coupling effect of parameters, and so forth. The starting point corresponded to 10 stimuli, 100 input nodes per one stimulus, 300 Hz stimulus spike frequency, and 3 Hz background noise (that corresponds to signal/noise ratio = 10). Effect of variation of different parameters is considered in the following subsections.

### 3.1. Informational Capacity

#### 3.1.1. Dependence of Conversion Quality on Number of Different Stimuli

**Figure 4:**
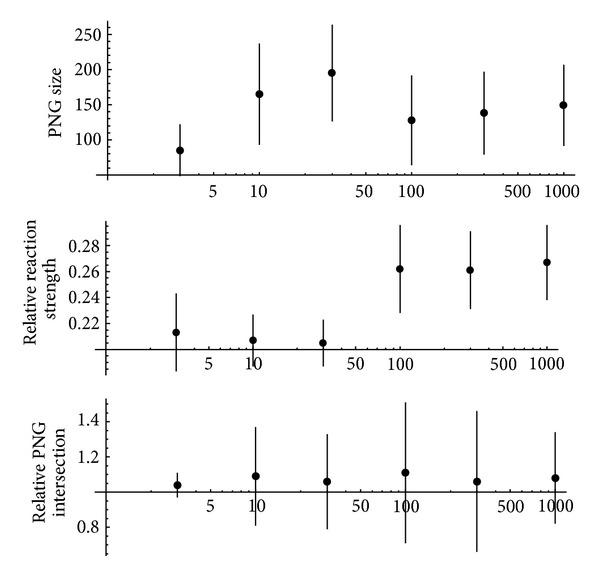
Conversion from rate/population coding to temporal coding in case of various numbers of different stimuli. The *x*-axis displays the number of stimuli using logarithmic scale. Length of the vertical lines drawn from the experimental points corresponds to standard deviation of the respective measured parameter.

The experiments were performed with number of different stimuli varying from 3 to 1000 (which is greater than the number of excitatory neurons in the network!). In all the experiments the conversion was successful; a PNG with selectivity equal to 1 was found for every stimulus. The detailed results for this experiment series are shown on Figure 4.

The most unexpected result is weak dependence of conversion quality on number of stimuli converted, even when there are more stimuli than excitatory neurons in the network. Average size of the polychronous groups performing the conversion was about 130. At least 20–25% of the respective PNG is activated after every presentation of the stimulus converted. All these PNGs are almost independent, although all points on the bottom plot are slightly above 1: it means that if a neuron belongs to some PNG it has a bit more chances to enter some other PNGs. Nevertheless, proximity of the average relative PNG intersection to 1 in all experiments is an indication that the network could convert successfully the number of stimuli significantly greater than the number of its neurons (however, in order to prove it experimentally much longer computation is required because computation time of the PNG detection algorithm described above is proportional to square of the number of different stimuli and even for 1000 stimuli the computation time was about 1.5 days).

### 3.2. Dependence of Conversion Quality on Stimulus Intensity

In case of uncorrelated presynaptic activity, LIF neuron behaves like a unit with sigmoid transfer function (with respect to spike rate). It is silent (in models without spontaneous firing) when presynaptic spikes are rare and fires with the maximum possible frequency determined by its refractory period in case of very frequent presynaptic spikes. Transfer between these “nothing” and “all” states may be more or less sharp: it depends on membrane potential decay time *τ* and average contribution of one presynaptic spike to membrane potential. In our case when *τ* is small (3 ms) and average synapse contribution is below 0.1 (while threshold membrane potential is set to 1), the sigmoid is rather similar to step function. For example, by decreasing stimulus spike frequency to 100 Hz, we observed that considerable number of stimulus presentations did not cause any network reaction. The same effect took place when we decreased number of input nodes per stimulus to 30. Naturally, under these conditions the network cannot operate as a converter. Probably, ability of the network to convert weaker stimuli could be facilitated by using more complex neuron models with threshold membrane potential adaptation [16] or based on homeostatic synaptic plasticity [17].

On the contrary, increasing intensity of stimuli due to enlarging subset of input nodes corresponding to one stimulus only improves the conversion quality. In experiments with 300 input nodes per stimulus all stimuli had PNGs with absolute selectivity, and reaction strength of these PNGs was significantly greater than that for 100 input nodes per stimulus, although some PNGs showed tendency to stick together under this condition. The corresponding data are gathered in Table 2.

### 3.3. Influence of Background Noise

In the last series of the experiments we varied level of background noise in the range 1 Hz–30 Hz. It was senseless to perform experiments with noise more intensive than 30 Hz because under this condition inhibition level in the network became insufficient and the network demonstrated ceaseless strong activity. The results of these experiments show that the conversion process is very stable with respect to noise. Although, naturally, conversion under condition of strong noise (30 Hz noise corresponds to the signal/noise ratio equal to 1) has lower quality in terms of PNG response strength and degree of PNG independence, but, nevertheless, for all stimuli in all experiments PNGs with absolute selectivity were found.

**Figure 5:**
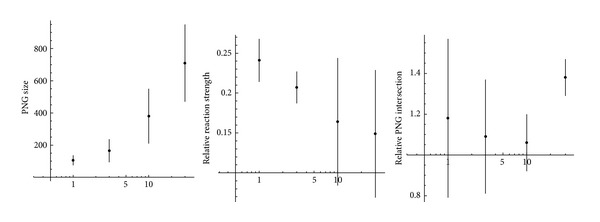
Conversion from rate/population coding to temporal coding under conditions of different background noise intensity (in Hz). It is displayed on the *x*-axes using logarithmic scale. Length of vertical lines corresponds to standard deviation of the respective parameter.

The respective experimental data are represented in Figure 5.

### 3.4. Randomization of Excitatory Connection Delays

**Figure 6:**
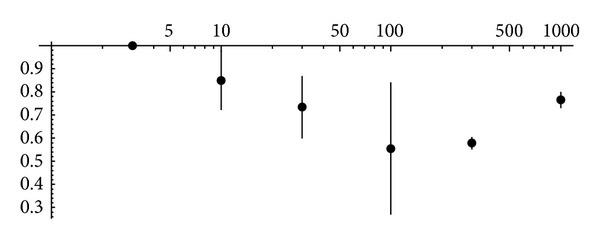
Part of stimuli for which PNGs with selectivity equal to 1 were found in case of SNN with randomized delays. The *x*-axis displays the number of stimuli using logarithmic scale. Each point corresponds to 10 experiments.

Probably, the most unexpected result obtained in this study is that synaptic plasticity was found to be unnecessary for achieving our goal. Indeed, the inventor of the term “polychronization”, Izhikevich, used synaptic plasticity (in fact, two kinds of it: long-term and short-term) in his experiments ([7] together with Szatmáry, [11], and others). Plasticity helped to highlight relatively rare neuron connections constituting PNGs in the ocean of other chaotic connections. We can hypothesize that since in our case the special selection of excitatory synaptic delays discussed above makes relative amount of PNGs much greater, it makes the positive effect of plasticity less important. In order to confirm this hypothesis, we performed experiments similar to ones considered above but with randomized values of delays in excitatory connections. Namely, after the network had been created using the rules described in the previous section, the delays of all its excitatory synapses were randomly permuted that made the network completely chaotic. Under these conditions, PNGs were detected but they lost their selectivity. To illustrate it quantitatively, we measured part of stimuli for which selective PNGs were found. Values of this parameter for different number of stimuli are shown in Figure 6. For each number of stimuli the experiment was repeated 10 times. We see that only the easiest experiment with 3 different stimuli was successful from the point of view of our selectivity criterion. It would be interesting to understand why the observed dependence is not monotonous but detailed exploration of properties of completely chaotic networks has no direct relation to main subject of this research.

Also, it should be noted that in this study we used the very simple simulation of input signal; it is possible that future research where we plan to work with more realistic sensory signals will require implementation of some forms of synaptic plasticity in my model. Indeed, the primary purpose of this study was to demonstrate how a simple homogenous SNN can convert signal from rate/population coding form to temporal code. But, if to consider this work in context of research efforts directed at simulation of integration and processing of multimodal real-world sensory information flows, then the next step should be creation of software model of sufficiently rich informational environment for the studied SNNs and reproduction of the reported results under these more realistic conditions. It would make possible incorporation of working memory mechanisms based on PNGs [7, 8] as the next layer of the whole SNN-based information processing system because these mechanisms assume temporal coding of stimuli memorized.

## 4. Conclusions

Thus, it was discovered in this work that under certain conditions chaotic and homogenous network consisting of simple LIF neurons can convert signal encoded using rate/population-based scheme to a form based on temporal coding. It is important because each of these two forms of information coding is very common for many (but different) parts of central nervous system. It is interesting that synaptic plasticity and learning are not required for successful recoding. Presence of global synchronizing signal propagated across the whole network is also not necessary.

In my approach the recoding process is considered as selective activation of a polychronous neuronal group specific for the given stimulus encoded using rate/population coding scheme. Therefore, it is essential that the network is enriched by potential PNGs due to special selection of propagation delays in excitatory interneuron connections; namely, these delays have values proportional to distances between the neurons as if they were placed at random points of imaginary sphere (see the details in [8]). Appropriate choice of numbers of inhibitory synapses for excitatory and inhibitory neurons, their weights, and propagation delays (see Table 1 and Figure 1) are also very significant, because inhibitory neurons play an important role in this construction; they should stop uncontrolled growth of excitation leading to permanent senseless activity of the network while permitting the pronounced network reaction to stimulus presentation (Figure 2).

In our approach, the selective PNGs are determined by a specially designed novel algorithm. It has linear complexity with respect to the main dimensions of the problem except the number of different stimuli (it has complexity proportional to square of this parameter).

The described above computational experiments confirmed that stable and quality conversion is performed by the described network in great range of stimuli parameters.

## Figures and Tables

**Table 1:** Parameters determining balance of excitation and inhibition in the network.

	Excitatory neurons	Inhibitory neurons
Amount	700	300

Maximum synaptic weight (for postsynaptic neurons)	0.19	10

Number of excitatory afferent synapses/total effective weight^1^	300/28.5	300/28.5

Number of inhibitory afferent synapses/total effective weight	10/50	30/150

Number of nonafferent excitatory synapses/total effective weight	100/9.5	100/9.5

Number of nonafferent inhibitory synapses/total effective weight	10/50	3/15

Synaptic propagation delays (for postsynaptic neurons), ms	2–10	1–3

^1^It is the mean weight multiplied by the number of synapses.

**Table 2:** Effect of increased stimulus intensity.

Number of input nodes per stimulus	PNG size	Relative PNG reaction strength	Relative PNG intersection
100	165 ± 72	0.207 ± 0.02	1.09 ± 0.28
300	121 ± 55	0.247 ± 0.12	1.15 ± 0.6
